# The Seven-State RF MEMS Miniaturized Broadband Reconfigurable Step Attenuator

**DOI:** 10.3390/mi15101182

**Published:** 2024-09-24

**Authors:** Yuheng Si, Siming Chen, Peifang Fu, Jian Yu, Binyi Ma, Qiannan Wu, Mengwei Li

**Affiliations:** 1 School of Instrument and Electronics, North University of China, Taiyuan 030051, China; 2 School of Instrument and Intelligent Future Technology, North University of China, Taiyuan 030051, China; 3 Academy for Advanced Interdisciplinary Research, North University of China, Taiyuan 030051, China; 4 Center for Microsystem Intergration, North University of China, Taiyuan 030051, China; 5 School of Semiconductors and Physics, North University of China, Taiyuan 030051, China; 6 Key Laboratory of Dynamic Measurement Technology, North University of China, Taiyuan 030051, China

**Keywords:** RF MEMS, thin film resistor, broadband, reconfigurable step attenuator

## Abstract

This paper presents a three-channel reconfigurable step attenuator based on radio frequency (RF) microelectromechanical system (MEMS) switches, in response to the current issues of high insertion loss and low attenuation accuracy of attenuators. The coplanar waveguide (CPW), cross-shaped power dividers, RF MEMS switches, and π-type attenuation resistor networks are designed as a basic unit of the attenuator. The attenuator implemented attenuation of 0~30 dB at 5 dB intervals in the frequency range of 1~25 GHz through two basic units. The results show that the insertion loss is less than 1.41 dB, the attenuation accuracy is better than 2.48 dB, and the geometric size is 2.4 mm × 4.0 mm × 0.7 mm. The attenuator can be applied to numerous fields such as radar, satellites, aerospace, electronic communication, and so on.

## 1. Introduction

An attenuator is one of the important components in RF microsystems and is a power-loss-type device. It has been widely used recently in microwave testing systems [1,2,3] and communication front-end modules [4,5,6] like spectrum analyzers, vector network analyzers [7], signal sources [8], and measurement instrument receivers [9] in military fields as radar [10], satellite [11], aerospace, communication [12,13,14], electronic warfare [15], and other fields, to achieve various functions such as reducing coupling, and controlling and adjusting signal power in micro devices [16,17,18,19,20].

In recent years, research on RF MEMS attenuators has been continuously conducted. There are different methods to study attenuators to implement their reconfigurability. Usually, people use attenuators with switches to achieve reconfigurable power control in the microwave range. For example, active switches like solid-state diodes [21], complementary metal oxide semiconductors (CMOSs) [22,23,24,25,26,27,28,29], and field-effect transistors [30] have been used to provide chip multi-bit step attenuation. However, these switches are not appropriate for application fields that demand large dynamic ranges and high attenuation accuracy due to their inherent high power consumption and low linearity, especially in the high-frequency range. Nevertheless, as for RF MEMS switches, they have the advantages of low insertion loss, large isolation, wide bandwidth, low power consumption, good linearity, good reliability, high integration, and small size, making them important components for forming excellent RF devices [31,32,33]. Due to their outstanding microwave performance, RF MEMS switches are considered as a promising component in miniaturized reconfigurable attenuators for testing instruments. 

In 2021, Wang et al. presented a high-dB-linear attenuator based on a variable gain amplifier that can operate from 6 to 18 GHz with a controlled variable gain range of 20 dB [34]. In 2022, Kim et al. proposed a DC-12 GHz 4-bit digital step attenuator that can achieve attenuation coverage of 30 dB with a least significant bit of 2 dB, insertion loss from 2.8 to 8.3 dB, and return loss less than 12 dB [35]. In 2023, a comb actuator was introduced into the attenuator, resulting in an attenuation dynamic range of 18 dB at 20 GHz, resulting in a more than 12-fold increase in linearity [36]. In the same year, a broadband, high-linearity 7-bit digital step attenuator was proposed with an insertion loss of 2.7 dB at 7 GHz [37]. In 2024, Quanzhen et al. presented an ultra-wideband, low-insertion-loss, and high-accuracy 6-bit digital step attenuator with an attenuation range of 31.5 dB in 0.5 dB steps, insertion loss better than 2.54 dB, and return loss better than −17 dB within 2–22 GHz [38]. However, there is currently relatively little research on RF MEMS attenuators, and their performance is not very satisfactory. Therefore, the problems of high insertion loss and low attenuation accuracy are still worthy of our research to improve these microwave characteristics [39,40,41,42,43,44].

In response to the above problems, this article proposes a three-channel step attenuator with RF MEMS switches, which can achieve step attenuation by controlling RF MEMS switches to select different attenuation resistance networks. The attenuator has the characteristics of wide bandwidth, low insertion loss, high accuracy, and small size.

## 2. Design and Simulation

### 2.1. Design of the Overall Attenuator Structure

**Figure 1:**
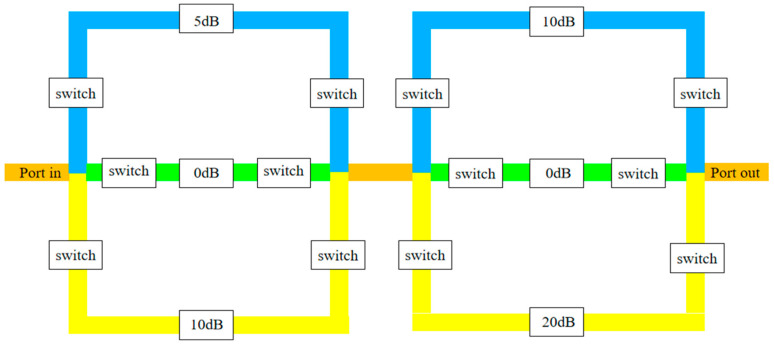
Structural diagram of attenuator.

This article presents a three-channel step attenuator based on RF MEMS switches, operating within 1~25 GHz. The goal of 0~30 dB attenuation in 5 dB steps is accomplished by controlling the MEMS switches to select different attenuation resistance networks. A one-section unit module consists of two power dividers, four RF MEMS switch groups, three attenuation networks, and signal lines. After the signal is input from the input end, different attenuation of the signal can be achieved by selecting the attenuation network above/below or the direct channel in the middle through MEMS switches. In order to achieve more attenuation states, this article uses a two-section three-channel attenuation unit to achieve seven attenuation states of 30 dB with a 5 dB step. The structural diagram is shown in Figure 1.

The values “0” and “1” represent whether different channels are gated; “0” represents that the signal does not pass through that channel, and “1” represents that the signal passes through that channel. Thus, the relationship between the attenuation amount and each attenuation path is obtained as shown in Table 1.

### 2.2. Design of MEMS Switch and Power Divider

The MEMS switches and power dividers are added between the input/output and attenuation network for signal flow. In MEMS attenuators, MEMS switches are key devices for channel selection to achieve different attenuation levels. Furthermore, MEMS switches directly affect the performance of the attenuators, such as the insertion loss and the isolation degree. Therefore, it is extremely essential to study MEMS switches. 

**Figure 2:**
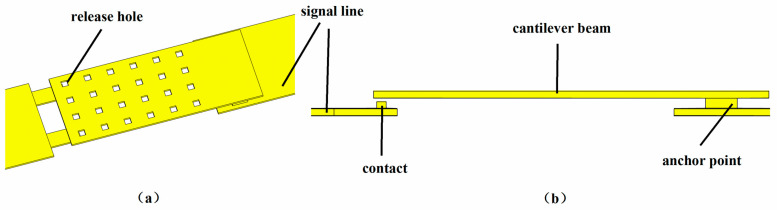
The structure of the MEMS switch. (**a**) Stereoscopic diagram; (**b**) cross section.

There are various upper electrode structures of MEMS switches, including polygonal, racket type, uniform meander structures [45,46,47,48], bending folding beam type [49], serpentine flexure [50], etc. However, in order to facilitate processing and production, as well as to be simple and practical, this article adopts a straight plate cantilever beam structure. The MEMS switch composed of a cantilever beam, contacts, anchor points, and release holes is shown in Figure 2. We can see that the surface of the upper electrode is neatly distributed with multiple release holes, allowing gas to flow out from both sides of the upper electrode and the release holes simultaneously, which can reduce the driving voltage and facilitate the release of the sacrificial layer. In order to better contact the upper and lower plates and improve the stability of the switches, a dual-contact structure is introduced.

The geometric parameters of the switch are shown in Table 2 below.

Driving electrodes are arranged below the cantilever beam of MEMS switches. When a certain driving voltage is applied, it can drive the pull-down of the cantilever beam. When the switches are in the off state, the switches will block the flow of microwave signal between the input and output ports, so as to minimize the transmission of the microwave signal to the output port as much as possible. When they are in a closed state, the microwave signal is transmitted from the input port to the output port along the switches.

The driving voltage is the minimum voltage required to achieve MEMS switch on/off. When the upper electrode is pulled to 2/3 of the up and down electrode distance, the electrostatic force and restoring force reach equilibrium, and the following formula can be derived:

(1) $$V=\sqrt{\frac{8{\mathrm{kg}}_{0}^{3}}{27\varepsilon_{0}A}}$$

(2) $$k=\frac{EWT^{3}}{4L^{3}}$$

Here, V is the driving voltage, k is the elasticity coefficient of the upper electrode, $g_{0}$ is the gap between the driving electrode and the upper electrode, $\varepsilon_{0}$ is the dielectric constant of the vacuum, and A is the facing area of two electrodes. E, W, T, and L are the Young’s modulus, width, thickness, and length of the cantilever, respectively.

From above, a theoretical estimate of the driving voltage for the switch can be calculated, which is approximately 20 V.

**Figure 3:**
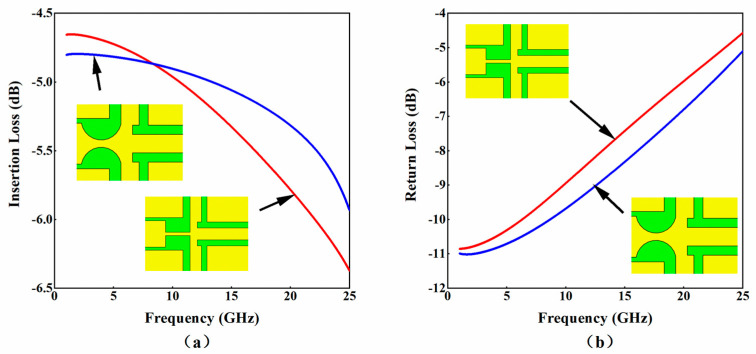
The simulation results in a cross-shaped power divider with a curved front end and a straight front end. (**a**) Insertion loss; (**b**) return loss.

The power divider is used to achieve impedance matching between the coplanar integrated waveguide (CPW) transmission line and the load, thereby reducing power loss and improving attenuation performance. Currently, the most commonly used power dividers for MEMS attenuators are cross-shaped power dividers. A comparison was made between the cross-shaped power divider with a curved front end used in this article and the commonly used cross-shaped power divider with a straight front end before designing the attenuator. Through simulation, the insertion loss and return loss of both can be obtained, as shown in Figure 3.

**Figure 4:**
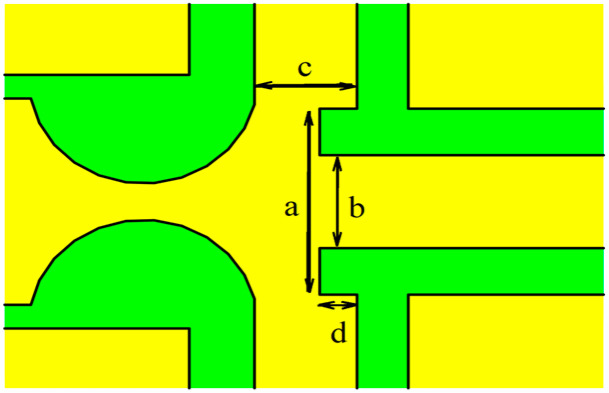
The structural details of the power divider.

In Figure 3, the red line represents a cross-shaped power divider with a straight front end, and the blue line represents a cross-shaped power divider with a curved front end. From the simulation results, it can be seen that the insertion loss of the two power dividers mentioned above is 6.38 and 5.93, and the return loss is 4.57 and 5.10, respectively. After comparison, the insertion loss of the cross-shaped power divider with a curved front end is relatively low, while the return loss is relatively high, so its microwave performance is the best by comparison. Therefore, in this article, the cross-shaped power divider with a curved front end is applied to the design of MEMS attenuators. The structural details and geometric parameters of the power divider are shown in Figure 4 and Table 3.

### 2.3. Design of the Attenuation Resistor Network

**Figure 5:**
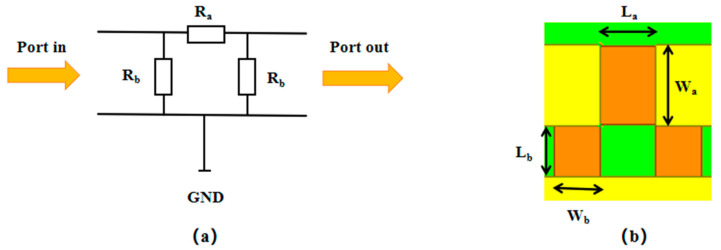
The structure of the attenuation resistance network. (**a**) Equivalent circuit; (**b**) structural diagram.

The function of the MEMS attenuator is for attenuation, so the attenuation resistor network is a necessary component of the attenuator. A π-type attenuation resistor network consisting of two parallel resistors and one series resistor is applied. The structure of the attenuation resistance network is shown in Figure 5. The attenuation resistance network can be equivalent to a two-port network. At low frequencies, the relationship of current and voltage between the input and output port can be obtained using the ABCD linear network parameters. At high frequencies, S-linear network parameters are utilized and an equivalence is established between S parameters and ABCD matrices to assess the performance of the proposed attenuator. The input and output impedance is the characteristic impedance of 50 Ω at high frequencies. For symmetric structures, S_21_ = S_12_ and S_11_ = S_22_. When impedance matches, S_11_ = 0.

The S-linear network parameters of the π-type attenuation resistance network are as follows:

(3) $$\left[\begin{array}{cc} S_{11} & S_{12}\\ S_{21} & S_{22} \end{array}\right]=\left[\begin{array}{cc} 0 & S_{12}\\ S_{21} & 0 \end{array}\right]$$

The ABCD linear network parameters of the π-type attenuation resistance network are as below:

(4) $$\left[\begin{array}{cc} A & B\\ C & D \end{array}\right]=\left[\begin{array}{cc} 1+\frac{R_{a}}{R_{b}} & R_{a}\\ \frac{2R_{b}+R_{a}}{R_{b}^{2}} & -1-\frac{R_{a}}{R_{b}} \end{array}\right]$$

The relationship between S-linear network parameters and ABCD linear network parameters can be transformed into the following formula:

(5) $$A=1+\frac{R_{a}}{R_{b}}=\frac{1+S_{21}^{2}}{2S_{21}}$$

(6) $$B=R_{a}=Z_{0}\cdot \frac{1-S_{21}^{2}}{2S_{21}}$$

(7) $$C=\frac{2R_{b}+R_{a}}{R_{b}^{2}}=\frac{1}{Z_{0}}\cdot \frac{1-S_{21}^{2}}{2S_{21}}$$

(8) $$D=-1-\frac{R_{a}}{R_{b}}=\frac{1+S_{21}^{2}}{2S_{21}}$$

Based on the above formula, the corresponding resistance value of the π-type attenuation resistance network can be derived, as follows:

(9) $$R_{a}=Z_{0}\cdot \frac{1-10^{-\frac{A}{10}}}{2\times 10^{-\frac{A}{20}}}$$

(10) $$R_{b}=Z_{0}\cdot \frac{-1-10^{-\frac{A}{20}}}{-1+10^{-\frac{A}{20}}}$$

In the formula, A is the attenuation amount; $Z_{0}$ is the port impedance, and its value is 50 Ω.

Therefore, the corresponding resistance value for each attenuation amount is obtained, as shown in Table 4 below.

The resistors that make up the attenuators are made by depositing thin layers of resistive material with appropriate geometry to absorb the correct percentage of electromagnetic wave energy according to the required attenuation.

The attenuation resistor uses a titanium thin film resistor. Titanium material has a low temperature coefficient of resistance and high reliability, making it an ideal thin film material. And there is relatively mature process technology, which is convenient for subsequent processing. The relationship between its resistance value and size is

(11) $$R=R_{s}\times \frac{L}{W}$$

In the formula, L is the length of the resistor, W is the width of the resistor, and $R_{s}$ is the square resistance value of the thin film resistor.

The corresponding resistance sizes for each attenuation amount are obtained consequently, as shown in Table 5 below.

## 3. Simulation and Results

**Figure 6:**
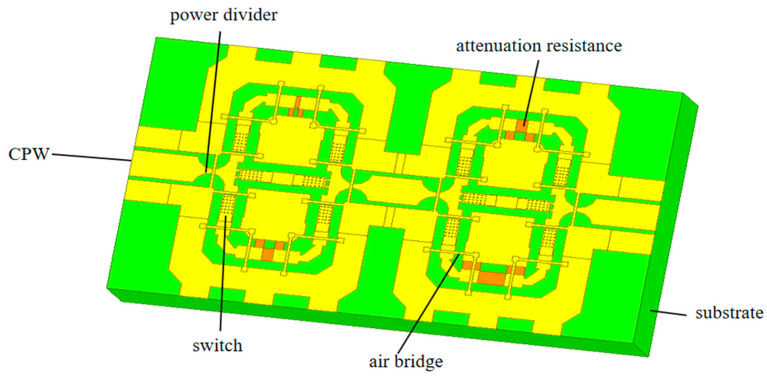
The structure of the attenuator.

The attenuator in this article is a two-section attenuator that uses CPW for signal transmission. Each unit module consists of cross-shaped power dividers, straight plate cantilever beam switches, a π-type attenuation resistor network, and signal lines. The first unit module consists of a 0 dB, 5 dB, and 10 dB attenuation resistor network; the second unit module consists of a 0 dB, 10 dB, and 20 dB attenuation resistor network, with an attenuation range of 0~30 dB and an attenuation step of 5 dB. The microwave performance of the attenuator within 1~25 GHz is simulated by Ansys software. The structure of the attenuator is shown in Figure 6. Owing to the excellent features of SiO_2_ such as stable chemical properties, high temperature resistance, and corrosion resistance, the 700 μm thick SiO_2_ material as substrate with a relative dielectric constant of 4.0 is applied in the attenuator. Due to its stable chemical properties, resistance to corrosion and oxidation, and diverse manufacturing methods, Au is used as a material for CPW transmission lines, with a conductivity of 4.09 × 10^7^ S/m.

In addition, to reduce the equivalent capacitance at the corners, various parts of the microstrip line are chamfered to compensate for the discontinuity effect caused by the right angle. In order to decrease signal loss during transmission, it is necessary to set the port impedance to 50 Ω to diminish the reflection of the signal at discontinuous interfaces and achieve impedance matching. The size parameters of the corresponding port of the CPW signal line can be calculated using ADS software, as shown in Table 6. The simulation result of the port impedance is basically consistent with the requirement.

After designing and optimizing the attenuator structure, simulation results of its RF performance can be obtained. In microwave systems, scattering parameters (S parameters) are usually used to characterize the performance of devices or systems. For MEMS attenuators, attenuation, insertion loss, and other important indicators that determine their excellent RF performance are discussed.

**Figure 7:**
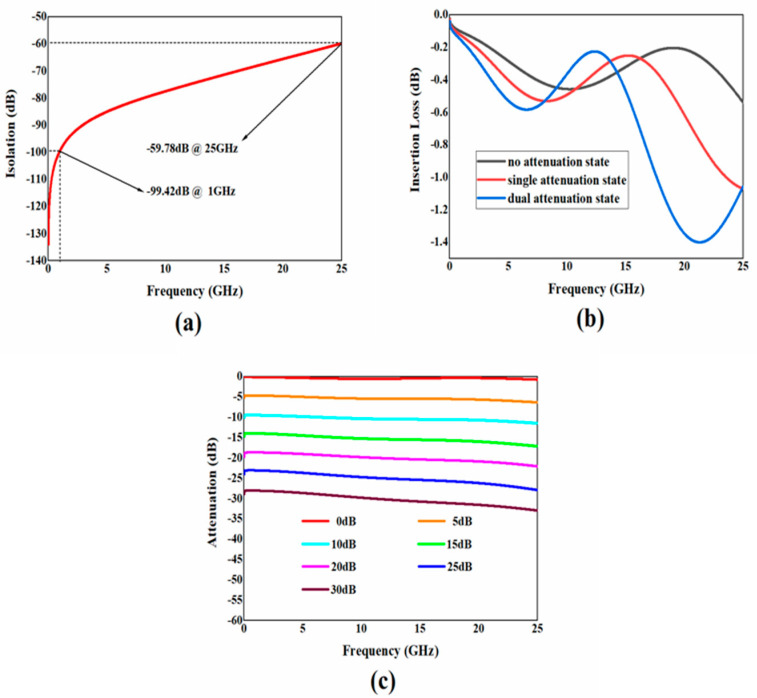
The simulation results of the RF performance of the attenuator. (**a**) Isolation; (**b**) insertion loss; (**c**) attenuation.

Within 1~25 GHz, different channels can be selected through switches to achieve attenuation of 0~30 dB in the step of 5 dB, which means a total of seven attenuation states can be achieved. The simulation results of the RF performance of the attenuator in various attenuation states are shown in Figure 7, and the specific situation is summarized in Table 7.

From Figure 7 and Table 7, it can be seen that within 1~25 GHz, a step attenuation of 5 dB can be achieved, with a maximum attenuation of 30 dB. It can be seen that the simulated attenuation value deviates slightly from the theoretical value. The reason is that there are parasitic capacitors and inductors at the junction between the attenuation resistor network and the microwave transmission line. The simulation result exhibits insertion loss less than 1.41 dB, return loss better than 13.21, isolation greater than 59.78 dB, and attenuation accuracy better than 2.48 dB within the entire frequency range. In the open state of the switches, the attenuator has a high degree of isolation, and there is less transmission of microwave signals to the output terminal. When the switches are in the closed state, the loss is relatively small, and the loss of microwave signals during transmission is also small. The results indicate that the attenuator has good RF characteristics and can be used for subsequent processing.

Compared with previous studies, this attenuator has achieved a wider bandwidth and smaller insertion loss through MEMS technologies, so the MEMS attenuator is worth more research. The comparison between this attenuator and other attenuators is shown in Table 8.

## 4. Processing and Manufacturing

**Figure 8:**
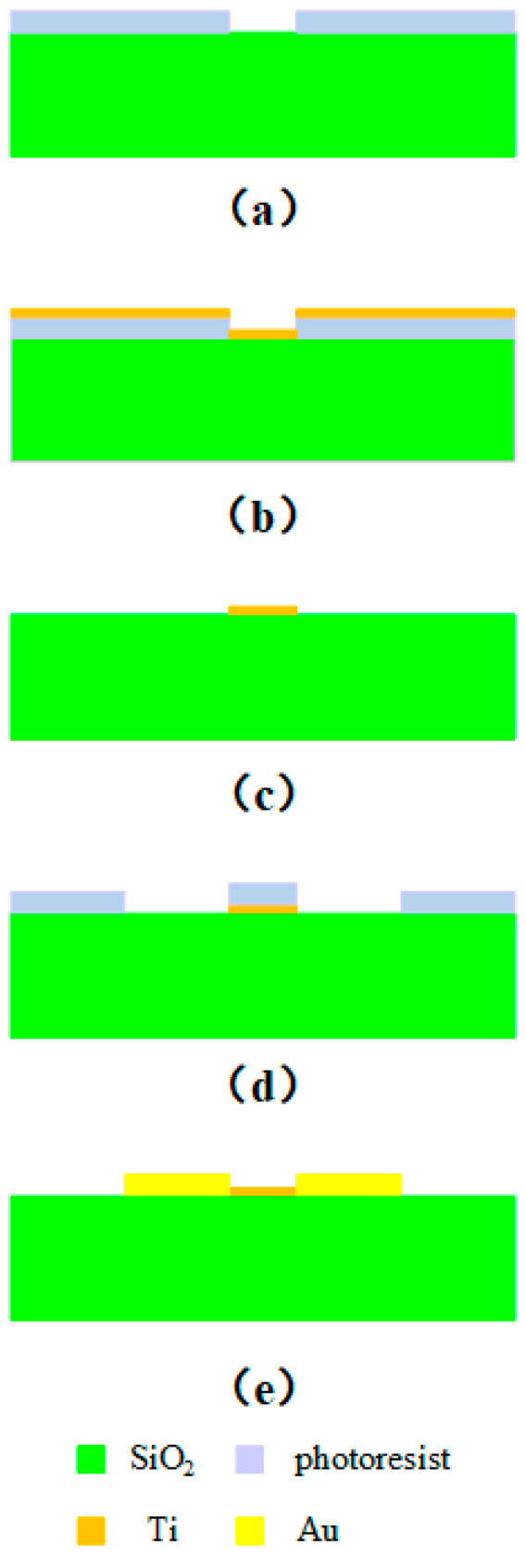
The manufacturing of titanium thin film: (**a**) development; (**b**) titanium thin film obtained by magnetron sputtering; (**c**) titanium thin film peeling; (**d**) CPW patterning; (**e**) electroplating Au to form CPW structure.

The manufacturing of titanium thin film belongs to reactive magnetron sputtering. The process flow diagram is shown in Figure 8, and the specific processing steps are as follows: (a) ultrasonically clean and pre-treat the substrate to improve the hydrophobicity of the wafer surface and increase its adhesion to the photoresist; spin coat the photoresist and pre-dry it, and then mid-dry it after photolithography to prevent the photoresist from completely dissolving in the developer during development; (b) fabricate the titanium thin film by magnetron sputtering; (c) dissolve the photoresist and lift off the titanium thin film; (d) sputter the seed layer and then glue, photoetch, and develop it to form CPW; (e) clean the wafer surface to increase its adhesion to Au, electroplate Au to the wafer surface to form a CPW transmission line, and remove the photoresist and seed layer.

By following the above steps, titanium thin film resistors can be produced.

**Figure 9:**
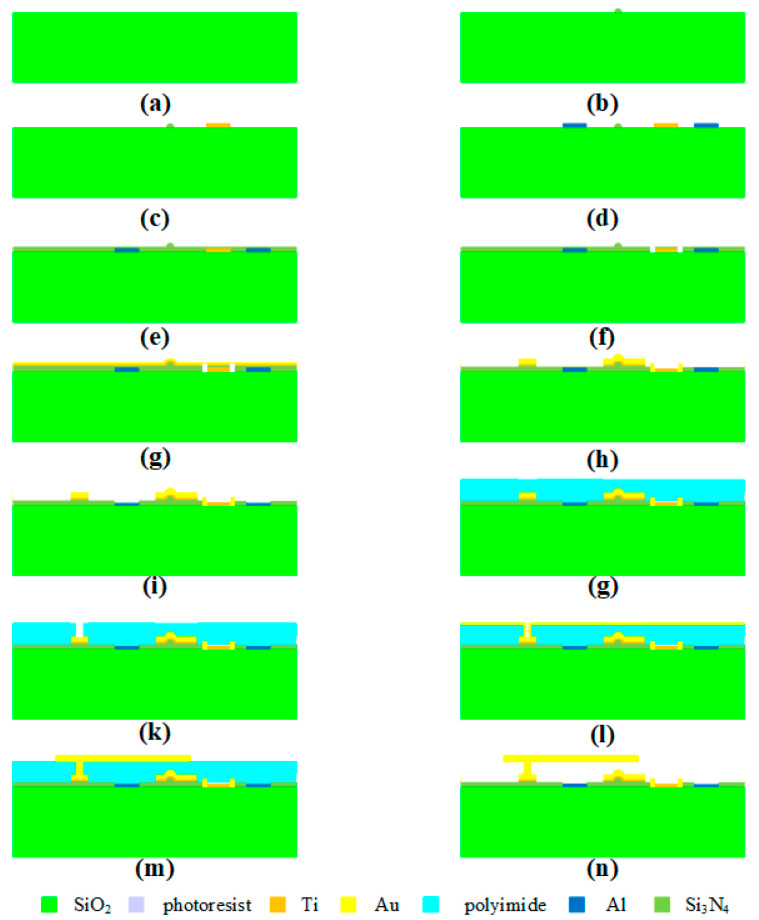
The flowchart of the MEMS step attenuator: (**a**) cleaning the surface of the wafer; (**b**) bump fabrication; (**c**) titanium thin film fabrication; (**d**) driving electrode fabrication; (**e**) isolation layer fabrication; (**f**) cascade fabrication; (**g**) seed layer fabrication; (**h**) CPW electroplating; (**i**) Pad windowing; (**j**) spin coating of sacrificial layer; (**k**) anchor point etching; (**l**) seed layer fabrication; (**m**) top electrode fabrication; (**n**) sacrificial layer releasing.

Based on the designed MEMS step attenuator, the process flow is designed, and the specific flowchart is shown in Figure 9. The process steps are as follows: (a) clean the surface of the wafer to remove impurities and ensure quality; (b) photoetch and clean the photoresist to fabricate an MEMS switch bump; (c) fabricate a titanium thin film; (d) use magnetron sputtering to fabricate an Al thin film, and strip it to obtain the driving electrode; (e) fabricate the isolation layer to prevent crosstalk between the DC signal caused by the driving voltage and the AC signal flowing through the CPW transmission line; (f) etch to cascade the titanium thin film resistor with the CPW transmission lines; (g) fabricate the seed layer; (h) electroplate the CPW transmission line; (i) add a Pad window to the driving electrode; (j) spin coat the sacrificial layer and pre-cure it; (k) etch the MEMS switch anchor point; (l) magnetron sputter the seed layer to make preparations for upper electrode electroplating; (m) electroplate to form the top electrode, and remove the photoresist and seed layer; (n) release the sacrificial layer.

Therefore, the manufacturing of the MEMS attenuator is completed.

## 5. Conclusions

The article introduces a three-channel reconfigurable step attenuator based on the basic unit module, including cross-shaped power dividers, RF MEMS switches, and π-type attenuation resistor networks. The attenuator realizes the attenuation of 0~30 dB in steps of 5 dB within 1~25 GHz through two basic units. The results show that within the entire frequency band, the insertion loss is less than 1.41 dB, the attenuation accuracy is better than 2.48 dB, and the geometric size is 2.4 mm × 4.0 mm × 0.7 mm. Through research, it has been found that the attenuator has excellent RF performance and has the potential to be processed and applied in fields such as electronic communication and satellite radar.

## Figures and Tables

**Table 1:** The relationship between the attenuation amount and each attenuation path.

Attenuation	The 1st-Section Unit Module			The 2nd-Section Unit Module		
	0 dB	5 dB	10 dB	0 dB	10 dB	20 dB
0 dB	1	0	0	1	0	0
5 dB	0	1	0	1	0	0
10 dB	1	0	0	0	1	0
15 dB	0	1	0	0	1	0
20 dB	1	0	0	0	0	1
25 dB	0	1	0	0	0	1
30 dB	0	0	1	0	0	1

**Table 2:** The geometric parameters of the switch.

Parameter	Value (μm)
length of cantilever beam	250
width of cantilever beam	100
thickness of cantilever beam	2
gap	3

**Table 3:** The geometric parameters of the power divider.

Parameter	Value (μm)
a	220
b	110
c	110
d	40
thickness	2

**Table 4:** The corresponding resistance value for each attenuation amount.

A/dB	R_a_/Ω	R_b_/Ω
5	30	178
10	71	96
20	248	61

**Table 5:** The corresponding resistance sizes for each attenuation amount.

A/dB	R_a_		R_b_	
	L_a_/μm	W_a_/μm	L_b_/μm	W_b_/μm
5	66	120	75	27
10	155	120	75	51
20	540	120	75	74

**Table 6:** The size parameters of the corresponding port of the CPW signal line.

Size Parameter	Value/μm
substrate height	700
gold thickness	2
signal line width	244
signal line length	4028
gap between signal lines	28

**Table 7:** The specific situation of the RF performance of the attenuator.

State	Attenuation (dB)		S_11_ (dB/@25 GHz)
	Average (dB)	Accuracy (dB)	
0 dB	0.39	0.38	13.21
5 dB	5.40	0.87	11.07
10 dB	10.38	1.03	9.87
15 dB	15.37	1.59	9.55
20 dB	20.11	1.85	9.50
25 dB	25.13	2.44	9.02
30 dB	30.45	2.48	6.82

**Table 8:** The comparison of the performance of the proposed attenuator with previous works.

Reference	Frequency (GHz)	Attenuation (dB)	Insertion Loss (dB)	Technology	Size
[22]	5~25	4	2.3	0.13 μm CMOS	180 × 60 μm^2^
[51]	5~15	1, 2, 4, 8, 16	5	SSI	-
[52]	DC~20	0~35, 5 (step)	1.7	MEMS	3.2 mm^2^
This work	1~25	0~30, 5 (step)	1.41	MEMS	2.4 × 4 mm^2^

## Data Availability

The data that support the findings of this study are available from the corresponding author upon reasonable request.
